# Structural analysis of the PATZ1 BTB domain homodimer

**DOI:** 10.1107/S2059798320005355

**Published:** 2020-05-29

**Authors:** Sofia Piepoli, Aaron Oliver Alt, Canan Atilgan, Erika Jazmin Mancini, Batu Erman

**Affiliations:** a Faculty of Engineering and Natural Sciences, Sabanci University, Orta Mahalle, Üniversite Caddesi No. 27, Orhanlı, Tuzla, 34956 Istanbul, Turkey; b School of Life Sciences, University of Sussex, Falmer, Brighton BN1 9QG, United Kingdom; c Sabanci University Nanotechnology Research and Application Center, SUNUM, 34956 Istanbul, Turkey

**Keywords:** BTB, POZ, PATZ1, transcription factors, co-repressors, dimerization interface, structure dynamics

## Abstract

PATZ1 is a ubiquitously expressed transcriptional repressor belonging to the ZBTB family that is functionally expressed in T lymphocytes. PATZ1 targets the *CD8* gene in lymphocyte development and interacts with the p53 protein to control genes that are important in proliferation and in the DNA-damage response. PATZ1 exerts its activity through an N-terminal BTB domain that mediates dimerization and co-repressor interactions and a C-terminal zinc-finger motif-containing domain that mediates DNA binding. Here, the crystal structures of the murine and zebrafish PATZ1 BTB domains are reported at 2.3 and 1.8 Å resolution, respectively. The structures revealed that the PATZ1 BTB domain forms a stable homodimer with a lateral surface groove, as in other ZBTB structures. Analysis of the lateral groove revealed a large acidic patch in this region, which contrasts with the previously resolved basic co-repressor binding interface of BCL6. A large 30-amino-acid glycine- and alanine-rich central loop, which is unique to mammalian PATZ1 amongst all ZBTB proteins, could not be resolved, probably owing to its flexibility. Molecular-dynamics simulations suggest a contribution of this loop to modulation of the mammalian BTB dimerization interface.

## Abstract 2

The crystal structures of the PATZ1 BTB domains from mammals and fish contain homodimers. The core dimer conformation of these BTB proteins is dynamically stable, despite the presence of highly flexible regions in the dimerization interface.

## 1. Introduction   

**Figure 1:**
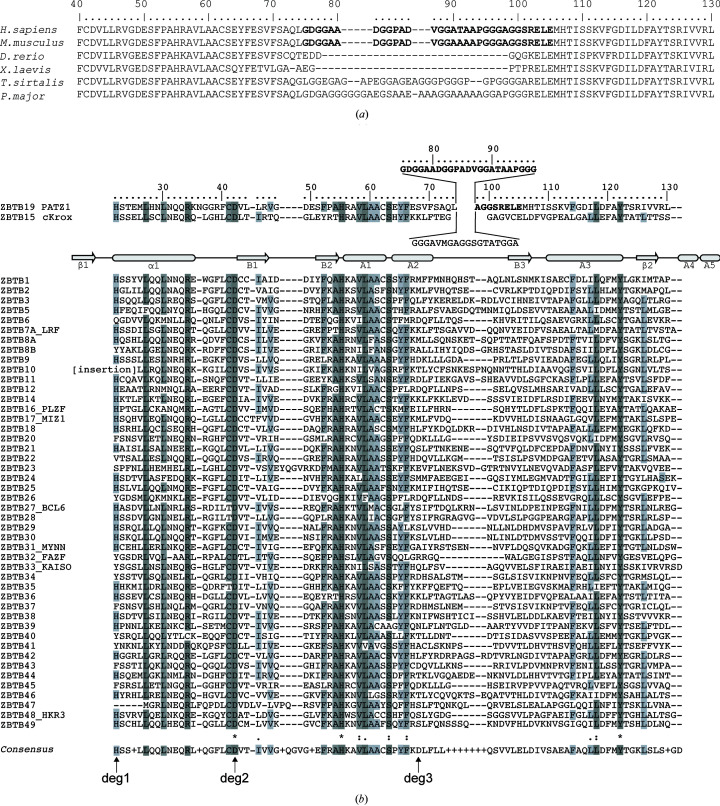
Sequence alignment of BTB domains of ZBTB transcription factors identifies a unique central region in PATZ1 that is conserved in mammals. (*a*) Sequence alignment of PATZ1 BTB domains from selected vertebrate species. The unique central sequence of the A2/B3 loop that is conserved in mammals and is missing in fish and amphibians is indicated in bold. (*b*) Sequence alignment of selected human ZBTB proteins and their predicted secondary structure. The sequences of the human PATZ1 and cKrox BTB domains with their unique extra region between the A2 helix and B3 strand are shown above. The PATZ1 amino acids that correspond to this region without electron-density assignments from the crystal structure are shown in bold. Arrows and rods identify predicted conserved β-strand and α-helical regions. The eight BTB domains with solved structures are annotated on the left with their common names in addition to the ZBTB nomenclature. Shading, asterisks, colons and periods identify conserved residues according to the *Clustal* format. A consensus sequence is shown at the bottom, with the three predicted degron residues involved in BTB domain stability indicated by arrows.

PATZ1 (POZ-, AT hook- and zinc-finger-containing protein 1), also known as ZBTB19, is a transcription factor that is present in all vertebrates (Fig. 1 ▸ *a*). It was first discovered in a yeast two-hybrid (Y2H) experiment, in which it associated through its BTB (broad complex, tramtrack and bric-a-brac) domain with the BTB domain of the transcription factor BACH2 (BTB and CNC homology 2; Kobayashi *et al.*, 2000 ▸). PATZ1 is also referred to as MAZR (Myc-associated zinc finger-related) because of the close similarity between its zinc-finger (ZF) domain and that of MAZ (Myc-associated zinc finger). While its expression can be detected in many cell types and developmental stages, PATZ1/ZBTB19/MAZR is highly expressed specifically in the early stages of T-lymphocyte differentiation, where it negatively regulates *CD8* gene expression (Bilic & Ellmeier, 2007 ▸). PATZ1 has been shown to participate in thymocyte development and CD4, CD8 and T_reg_ lineage choice by repressing the expression of ThPOK (ZBTB7B/ZBTB15/cKrox), another BTB domain-containing transcription factor (Sakaguchi *et al.*, 2010 ▸, 2015 ▸; He *et al.*, 2010 ▸), and the expression of the FOXP3 transcription factor (Andersen *et al.*, 2019 ▸). The functions of PATZ1 are however not limited to lymphocytes as its expression is ubiquitous. An early embryonic role for PATZ1 has been suggested, as PATZ1^−/−^ mice are embryonic lethal or born at non-Mendelian frequency and are small in size depending on the genetic background (Sakaguchi *et al.*, 2010 ▸). PATZ1 also negatively regulates induced pluripotent stem-cell (iPSC) generation (Ow *et al.*, 2014 ▸; Ma *et al.*, 2014 ▸). This function may be related to its interaction with the p53 tumour suppressor, as demonstrated by various studies (Valentino, Palmieri, Vitiello, Pierantoni *et al.*, 2013 ▸; Keskin *et al.*, 2015 ▸; Chiappetta *et al.*, 2015 ▸).

Structurally, PATZ1 belongs to the POZ (pox virus and zinc finger) or ZBTB (zinc finger and BTB) family of transcription factors (Lee & Maeda, 2012 ▸). Proteins belonging to this family have been implicated in many biological processes, including transcriptional regulation and development (Chevrier & Corcoran, 2014 ▸), whilst their dysfunction in vertebrates has been linked to tumorigenesis. ZBTB proteins bind to DNA through their ZF domains, and use their BTB domains for oligomerization (Bonchuk *et al.*, 2011 ▸) and the recruitment of co-repressors and chromatin-remodelling factors (Bardwell & Treisman, 1994 ▸; Siggs & Beutler, 2012 ▸). The human genome encodes 49 members of the ZBTB family (Fig. 1 ▸ *b*), all of which contain an N-terminal BTB domain and a variable number of ZF motifs at their C-terminus. Two members of this family contain an additional motif in the form of an AT hook (PATZ1 and PATZ2/ZBTB24).

The BTB domain is a structural feature that mediates functional interactions between proteins (Perez-Torrado *et al.*, 2006 ▸). Most of the available BTB domain structures from the ZBTB family are homodimers formed by the assembly of two identical monomers (Ahmad *et al.*, 1998 ▸, 2003 ▸; Li *et al.*, 1999 ▸; Schubot *et al.*, 2006 ▸; Stogios *et al.*, 2007 ▸, 2010 ▸; Ghetu *et al.*, 2008 ▸; Stead *et al.*, 2008 ▸; Cerchietti *et al.*, 2010 ▸; Sakamoto *et al.*, 2017 ▸; McCoull *et al.*, 2017 ▸, 2018 ▸; Kamada *et al.*, 2017 ▸; Yasui *et al.*, 2017 ▸; Kerres *et al.*, 2017 ▸; Cheng *et al.*, 2018 ▸). In the case of Myc-interacting zinc-finger protein 1 (MIZ1), the structures of both a homodimer (Stogios *et al.*, 2010 ▸) and a homotetramer (Stead *et al.*, 2007 ▸) have been reported.

The overall fold of the BTB domain is highly conserved, containing the following secondary-structure elements: β1–α1–B1–B2–A1–A2–B3–A3–β2–A4–A5 (Stogios *et al.*, 2005 ▸). A unique feature of the PATZ1 BTB domain is a long glycine- and alanine-rich central loop between A2 and B3 (Kobayashi *et al.*, 2000 ▸; Fig. 1 ▸). This central loop is conserved in all vertebrate PATZ1 proteins, but it is absent in those from fish and amphibians. The two BTB monomers are known to homodimerize through a specific dimerization interface that includes β1–α1, A1–A2 and β2–A5. Although homodimerization seems to be favoured, heterodimeric interactions between pairs of ZBTB proteins have also been documented. The crystal structure of a tethered ‘forced’ heterodimer between the BTB domains of MIZ1 and B-cell lymphoma 6 protein (BCL6) suggests that heterodimers can use the same interface as homodimers (Stead & Wright, 2014 ▸). Together with BCL6, MIZ1 seems to be a promiscuous member of the ZBTB family, making more heterodimers than any other BTB domain. MIZ1 functionally interacts with BCL6 in germinal centre B cells (Phan *et al.*, 2005 ▸), ZBTB4 (Weber *et al.*, 2008 ▸), ZBTB36 (Lee *et al.*, 2012 ▸), ZBTB49 (Jeon *et al.*, 2014 ▸) and NAC1 (nucleus accumbens-associated 1; Stead & Wright, 2014 ▸). PATZ1 can also form heterodimers with other BTB-domain-containing proteins such as PATZ2 (Huttlin *et al.*, 2015 ▸), BACH1 and BACH2 (Kobayashi *et al.*, 2000 ▸).

The mechanism controlling the homodimerization versus heterodimerization of BTB domains has not been elucidated. Co-translational dimerization, a mechanism often required in protein-complex assembly, may be at play (Kramer *et al.*, 2019 ▸). Recently, a dimerization quality-control mechanism for BTB proteins has been proposed in which the stability of a homodimer would exceed that of heterodimers because of the structural masking of destabilizing residues (Herhaus & Dikic, 2018 ▸; Mena *et al.*, 2018 ▸). According to this model, the preferential exposure of three ‘degron’ residues on BTB heterodimers results in targeting by ubiquitin ligases and a shorter half-life. Whether this mechanism is universally shared by all BTB domain-containing proteins, including members of the ZBTB family, remains unclear.

Homodimerization of BCL6 and promyelocytic leukaemia zinc-finger (PLZF) proteins creates a charged groove that binds nuclear receptor co-repressors such as NCOR1, NCOR2 (SMRT) and BCOR (Huynh & Bardwell, 1998 ▸; Wong & Privalsky, 1998 ▸; Huynh *et al.*, 2000 ▸; Melnick *et al.*, 2000 ▸, 2002 ▸). NCOR1 and SMRT are structurally disordered proteins that share 45% identity (Granadino-Roldán *et al.*, 2014 ▸) and contain a conserved 17-amino-acid BCL6-binding domain (BBD). These co-repressors are components of large complexes containing histone deacetylases (Li *et al.*, 2000 ▸) that contribute to transcriptional silencing. It is not known whether co-repressor binding is a generalizable feature of BTB homodimers, as MIZ1, FAZF and LRF BTB homodimers do not interact with these co-repressors (Stogios *et al.*, 2007 ▸, 2010 ▸). The PATZ1 BTB domain has been shown to interact with NCOR1; however, it is not known whether the inter­action is mediated by an interface similar to that of BCL6 and PLZF (Bilic *et al.*, 2006 ▸).

Prior to this work, the structures of eight different proteins belonging to the ZBTB family were available in the Protein Data Bank (PDB): LRF/Pokémon (ZBTB7A), PLZF (ZBTB16), MIZ1 (ZBTB17), BCL6 (ZBTB27), MYNN (ZBTB31), FAZF (ZBTB32), KAISO (ZBTB33) and HKR3/TZAP (ZBTB48). In order to obtain biological insights into the binding of PATZ1 to co-repressors, we determined the atomic structure of the PATZ1 (ZBTB19) BTB domain. To probe the role of the unique A2/B3 central loop, we obtained crystal structures of both the mouse PATZ1 BTB domain and its zebrafish orthologue.

## 2. Materials and methods   

### 2.1. Protein expression and purification   

The human isoform 3 (Q9HBE1-3) and mouse (Q9JMG9) PATZ1 BTB sequences are 98.9% identical (as determined using *LALIGN*; Gasteiger *et al.*, 2003 ▸), diverging at only one residue (T91A). The mouse PATZ1 BTB protein-coding sequence (12–166) was PCR-amplified from a CMV-HA plasmid construct (Keskin *et al.*, 2015 ▸) containing the full-length mouse PATZ1 cDNA and cloned into a pET-47b bacterial expression plasmid (Novagen) between SmaI and NotI restriction sites in frame with an N-terminal 6×His tag and an HRV 3C protease cleavage site for fusion-tag removal. The resulting plasmid was transformed into the *Escherichia coli* Rosetta 2(DE3) strain and grown at 310 K by shaking at 180 rev min^−1^ in Terrific/Turbo Broth (TB) medium supplemented with 50 µg ml^−1^ kanamycin and 33 µg ml^−1^ chloram­phenicol until the absorbance at 600 nm reached a value of 0.6. Expression of the fusion protein was induced by the addition of 0.1 m*M* isopropyl β-d-1-thiogalactopyranoside (IPTG) and growth was continued for 16 h at 291 K. The zebrafish (*Danio rerio*) PATZ1 BTB protein-coding sequence (1–135) was PCR-amplified from zebrafish genomic DNA (a kind gift from Dr S. H. Fuss), cloned and expressed in the same bacterial expression plasmid as described above.

The cells were harvested by centrifugation, resuspended in 25 ml lysis buffer (50 m*M* HEPES pH 7, 250 m*M* NaCl, 10 m*M* imidazole, 0.5 m*M* TCEP, DNase and protease-inhibitor cocktail) and disrupted by sonication on ice. The lysate was clarified by centrifugation at 26 700*g* for 45 min at 277 K. The supernatant was applied onto a HisPur Cobalt Resin column (Thermo Fisher) previously equilibrated with wash buffer (50 m*M* HEPES, 250 m*M* NaCl, 0.5 m*M* TCEP, 10 m*M* imidazole). Following a 10 min incubation at 227 K and the application of wash buffer, the protein was then eluted by the addition of elution buffer (50 m*M* HEPES, 250 m*M* NaCl, 0.5 m*M* TCEP, 300 m*M* imidazole). The collected eluate was concentrated to 2.6 mg ml^−1^ using a Sartorius Vivaspin 20 column (10K molecular-weight cutoff) for additional purification by size-exclusion chromatography (SEC) using a HiLoad 16/600 Superdex 75 prep-grade column (GE Healthcare) in gel-filtration buffer (20 m*M* HEPES, 250 m*M* NaCl, 0.5 m*M* TCEP) at 277 K. Fractions were analyzed on a 14% SDS–PAGE gel by electrophoresis and those containing PATZ1 BTB were pooled and concentrated to 9 mg ml^−1^. The zebrafish PATZ1 BTB domain (69% sequence identity to mouse BTB) was expressed and purified as described above for the mouse BTB domain. The final concentration of the protein was 8.5 mg ml^−1^.

### 2.2. Crystallization   

All crystallization experiments were performed at 291 K using the sitting-drop vapour-diffusion method. The initial screening of 768 conditions was performed at the University of Sussex crystallization facility using a Crystal Phoenix dispensing robot to pipette 0.1 µl protein solution and 0.1 µl precip­itant solution into single drops in 96-well plates. Crystals of the mouse PATZ1 BTB domain appeared after 72 h in 0.1 *M* MMT (dl-malic acid, MES monohydrate, Tris), 25%(*w*/*v*) PEG 1500. Crystals of the zebrafish PATZ1 BTB domain appeared after 72 h in 40%(*v*/*v*) PEG 500 MME, 20%(*w*/*v*) PEG 20K, 0.1 *M* Tris pH 8.5, 0.06 *M* magnesium chloride hexahydrate, 0.06 *M* calcium chloride dihydrate.

### 2.3. Data collection and processing   

For data collection, single crystals were briefly immersed in mother liquor supplemented with 20% glycerol prior to flash-cooling in liquid nitrogen. For mouse PATZ1 BTB, data were collected to 2.29 Å resolution on beamline I04 at Diamond Light Source, Didcot, UK. The diffraction data were indexed, integrated, scaled and reduced with *xia*2 (Winter, 2010 ▸) and *AIMLESS* (Evans & Murshudov, 2013 ▸). The space group was *P*4_1_2_1_2 (unit-cell parameters *a* = *b* = 43.23, *c* = 162.95 Å, α = β = γ = 90°), with one molecule in the asymmetric unit.

For zebrafish PATZ1 BTB, data were collected to 1.8 Å resolution on beamline I04 at Diamond Light Source. The diffraction data were indexed, integrated, scaled and reduced with *xia*2 and *XDS* (Kabsch, 2010 ▸). The space group was *P*3_1_21 (unit-cell parameters *a* = *b* = 43.08, *c* = 123.64 Å, α = β = γ = 90°), with one molecule in the asymmetric unit.

Detailed X-ray data-collection and refinement statistics are given in Table 1 ▸.

### 2.4. Structure solution and refinement   

The structure of mouse PATZ1 BTB was solved by molecular replacement with *Phaser* (McCoy *et al.*, 2007 ▸) using the PLZF BTB structure (PDB entry 1buo; Ahmad *et al.*, 1998 ▸) as a search template. The identified solution was then subjected to rounds of manual rebuilding with *Coot* (Emsley *et al.*, 2010 ▸) and refinement with *Phenix* (Liebschner *et al.*, 2019 ▸) to give final *R*_work_ and *R*_free_ factors of 20.9% and 24.5%, respectively. The final structure was validated with *MolProbity* (Williams *et al.*, 2018 ▸) and deposited together with the structure factors in the Protein Data Bank as entry 6guv.

The structure of zebrafish PATZ1 BTB was solved by molecular replacement with *Phaser* using the mouse PATZ1 BTB structure (PDB entry 6guv) as the search template. The identified solution was then subjected to rounds of manual rebuilding with *Coot* and refinement with *Phenix* to give final *R*_work_ and *R*_free_ factors of 21.5% and 22.4%, respectively. The final structure was validated with *MolProbity* and deposited together with the structure factors in the Protein Data Bank as entry 6guw.

Detailed X-ray data-refinement statistics are given in Table 1 ▸.

### 2.5. Sequence and structure analysis   

The sequences of PATZ1 proteins from different organisms were retrieved from the NCBI Reference Sequence Database (RefSeq; Pruitt *et al.*, 2007 ▸). Except for *Homo sapiens* (NP_114440.1) and *Mus musculus* (NP_001240620.1) for mammals, one organism only was chosen for each group of different species of vertebrates: *Danio rerio* (XP_009300883.1) for fish, *Xenopus laevis* (XP_018117120.1) for amphibians, *Thamnophis sirtalis* (XP_013922905.1) for reptiles and *Parus major* (XP_015499085.1) for birds. Sequence limits were determined based on the annotations of the BTB domain in the UniProt database (The UniProt Consortium, 2019 ▸). The 49 members of the human ZBTB protein family were retrieved from Swiss-Prot and a multiple sequence alignment was obtained using the *PROMALS*3*D* online tool (Pei *et al.*, 2008 ▸). This alignment incorporates the structural information from the available PDB structures of these proteins. The secondary-structure nomenclature refers to that of Stogios *et al.* (2005 ▸) (Figs. 1 ▸ *b*, 2 ▸ *a*, 2 ▸ *c* and 2 ▸ *f*). ZBTB4 was excluded from this alignment because of its N-terminal serine-rich repetitive insertions, which are beyond the scope of this description. Shading was added according to the percentage of similarity for every position in the alignment visualized in *Jalview* 2 (Waterhouse *et al.*, 2009 ▸) that generated a consensus sequence. A list of residues involved in homodimer interaction interfaces was obtained by *PDBePISA* (Krissinel & Henrick, 2007 ▸) and was graphically rendered on the protein structure with *VMD* (Humphrey *et al.*, 1996 ▸). Intra-chain and inter-chain inter­actions were retrieved by *PIC* (*Protein Interactions Calculator*; Tina *et al.*, 2007 ▸) and the *VMD* *Salt Bridges* and *Timeline* plugins. Structural alignments were calculated using the *MultiProt* server (Shatsky *et al.*, 2004 ▸).

### 2.6. Modelling   

The central loop of the mammalian PATZ1 BTB structure (residues 83–113) was modelled as a monomer using the *PRIMO* suite (Hatherley *et al.*, 2016 ▸) based on the *MODELLER* program (Šali & Blundell, 1993 ▸). Structural information from *MEME* motif analysis of the loop sequence was added to the template (Bailey & Elkan, 1994 ▸). The obtained model was aligned with the deposited structure (PDB entry 6guv) in *PyMOL* (version 1.8; Schrödinger). The coordinates of the loop model were added to the crystal structure and fragments were joined using the *VMD AutoPSF* plugin. *SymmDock* (Schneidman-Duhovny *et al.*, 2005 ▸) was used to reconstruct the dimer conformation.

The stability of the new structure was tested by molecular-dynamics (MD) simulations in *NAMD* (Phillips *et al.*, 2005 ▸). The protein structure model was centred in a solvent box built according to the protein size and padded with at least a 10 Å layer of water in every direction. The solvent was modelled explicitly using TIP3W water molecules. 0.15 *M* KCl was added to ionize the solvent. The MD simulation was performed using the CHARMM27 force field (Brooks *et al.*, 2009 ▸) in *NAMD*. Periodic boundary conditions were applied in which long-range electrostatic interactions were treated using the particle mesh Ewald method (Darden *et al.*, 1999 ▸) and the cutoff distance was set to 12 Å. All simulations were run as duplicates at a constant temperature of 310 K for at least 200 ns.

In one of the MD simulations of human PATZ1 BTB, the flexible loop region in one of the monomers formed an extra β-strand structure. The model was further refined by once more duplicating this monomer into the dimeric form. This refinement was processed by taking a frame from the run in which the root-mean-square deviation (r.m.s.d.) with the initial structure, excluding the loop, was minimal (3.04 Å) and by mirroring the information from the monomer with the formed secondary structures to the other monomer with *M-ZDOCK* (Pierce *et al.*, 2005 ▸), recreating the dimer by symmetry. The stability of the new structure was confirmed by additional MD simulations (twice, 200 ns each). *ModLoop* (Fiser & Sali, 2003 ▸) was used to model the coordinates of the missing residues (70–76) in the zebrafish PATZ1 BTB structure (PDB entry 6guw) and the stability of the modelled structure was assessed by MD as before.

## 3. Results and discussion   

### 3.1. Structural features of the murine and zebrafish PATZ1 BTB domains   

**Figure 2:**
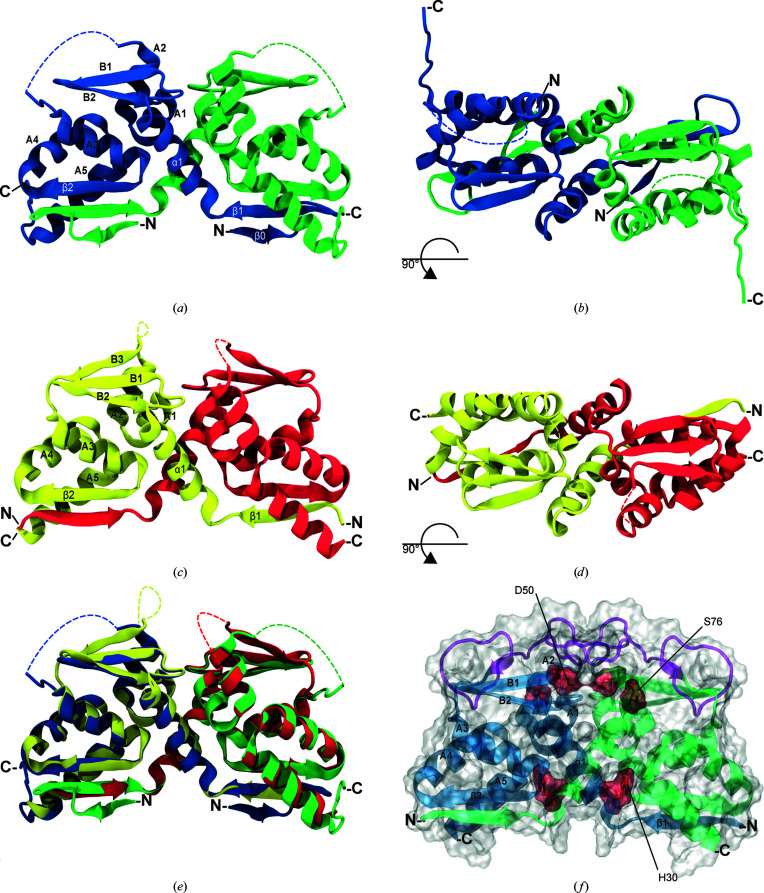
Structure of the PATZ1 BTB domain. (*a*) Crystal structure of the BTB domain of the mouse PATZ1 protein (PDB entry 6guv) in cartoon representation (front view). The crystallographic asymmetric unit contains a PATZ1 BTB domain monomer (blue) with a second monomer (green) created by crystallographic symmetry. Secondary structures are indicated in capital and Greek letters. (*b*) Top view of the mouse PATZ1 BTB domain structure. N- and C-­termini are indicated on one monomer. The coordinates of 31 residues in a central region, unique to mammalian PATZ1 BTB domains, could not be assigned (indicated by a dotted loop). (*c*) Crystal structure of the BTB domain of the zebrafish PATZ1 protein (PDB entry 6guw) with individual monomers coloured yellow and red (front view). (*d*) Top view of the zebrafish PATZ1 BTB structure. The coordinates of seven residues in the zebrafish PATZ1 BTB domain could not be assigned (indicated by a dotted loop). (*e*) Superimposition of the mouse (blue and green) and zebrafish (yellow and red) PATZ1 BTB domains (r.m.s.d. of 0.62 Å). (*f*) A space-filling representation of the mouse PATZ1 BTB domain structure. The predicted structure (in purple) of the central region was generated by homology modelling followed by conformation equilibration using MD simulations. Note that the modelled structure contains a predicted short β-strand. The three conserved degron residues, annotated at the bottom of Fig. 1 ▸(*b*) and predicted to play a role in BTB dimer degradation, are highlighted in red. Numbering refers to the residues in the crystal structure.

Here, we report the crystal structures of the BTB domains of murine and zebrafish PATZ1 (Fig. 2 ▸). In a similar way to other members of the ZBTB family, both PATZ1 BTB crystal structures reveal a strand-exchange homodimer, organized as a core fold BTB domain preceded by an N-terminal extension that interacts with the partner chain in the dimer. The characteristic secondary structures of the dimerization interfaces (β1–α1, A1–A2 and β2–A5) are conserved. Size-exclusion chromatography data for both the murine and zebrafish BTB domains suggest that the homodimeric complex is the most abundant oligomerization state found in solution (Supplementary Fig. S1). The murine PATZ1 BTB domain protein was expressed from a construct encoding amino acids 12–166 preceded at the N-terminus by 20 amino acids comprising a His tag and an HRV-3C protease digestion site (Figs. 2 ▸ *a* and 2 ▸ *b*). The last ten of these amino acids (ALEVLFQGPG) are visible in the structure and fold into a β-strand (β0) antiparallel to the first N-terminal PATZ1 β-strand (β1) (Supplementary Fig. S2). Superposition of this structure with that of BCL6 BTB in complex with co-repressor peptides (PDB entries 1r2b and 3bim; Ahmad *et al.*, 2003 ▸; Ghetu *et al.*, 2008 ▸) suggests that these extra residues structurally mimic the β-strand-forming residues in both SMRT and BCOR (Supplementary Fig. S3), although their amino-acid sequence is not conserved. Attempts to crystallize the mouse PATZ1 BTB construct following cleavage of the His tag failed, suggesting that the extra N-terminal amino acids are likely to aid the crystallization process in this case.

The mammalian PATZ1 (ZBTB19) protein is predicted to contain a 31-amino-acid A2/B3 loop (residues 75–105) that is partially conserved in cKrox (ZBTB15), which sets PATZ1 apart from the other ZBTB family members (Figs. 1 ▸ *a* and 1 ▸ *b*). This large loop replaces a shorter amino-acid stretch that forms a β-strand (B3) in other ZBTB family proteins, as described in detail by Stogios *et al.* (2005 ▸). This loop is glycine- and alanine-rich and is predicted to be partially disordered; however, a short β-strand is predicted for the last six residues. The single difference between the human and mouse PATZ1 BTB domains (T91A) is found within this large loop. In the crystal structure no density could be assigned to the residues belonging to the A2/B3 loop, suggesting that these amino acids are partially disordered or flexible.

Interestingly, in the mouse PATZ1 BTB structure a seven-amino-acid stretch from the C-terminus of an adjacent molecule in the crystal unit cell extends into the region normally occupied by the B3 β-strand in other ZBTB proteins (Supplementary Fig. S4). This crystallization artefact presumably aids crystal packing, as the ‘B3 strand mimic’ appears to stabilize the β-sheet formed by B1 and B2. To test this hypothesis, we crystallized a mouse PATZ1 BTB construct lacking the last seven C-terminal amino acids. The crystals of this protein diffracted poorly and to lower resolution (3.4 Å), showing the same homodimeric BTB domains in a different crystal packing (data not shown). This suggests that these seven amino acids were in fact important for stabilizing the B1–B2 β-sheet and for crystal packing (hence the better diffraction), yet their absence did not encourage the folding of the A2/B3 loop.

**Figure 3:**
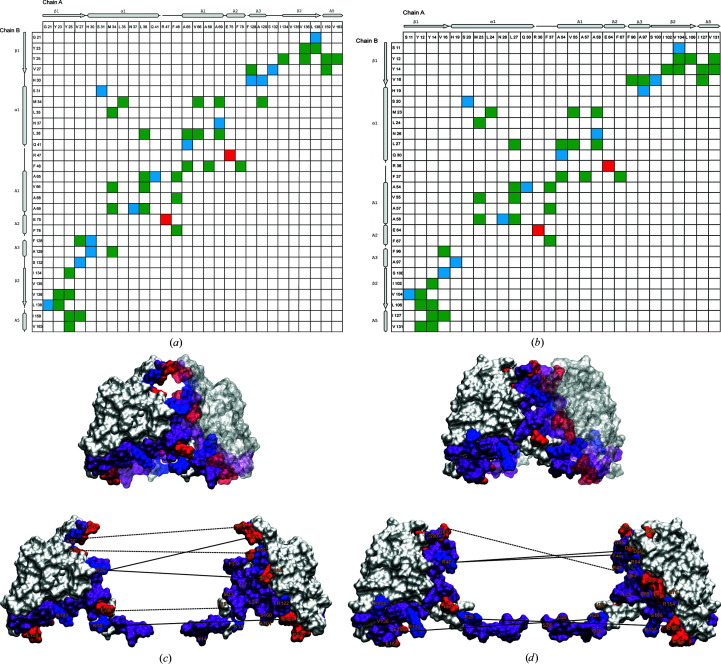
Identification of residues involved in homodimer interaction interfaces. Contact maps of inter-chain residue interactions of the mouse (*a*) and zebrafish (*b*) PATZ1 BTB domain crystal structures. Hydrophobic interactions (green), hydrogen bonds (blue) and ionic bonds (red) are indicated. The relevant elements of the secondary structures are shown for orientation. A highly charged dimerization interface mediates PATZ1 BTB homodimerization. (*c*) A split homodimer view in surface representation and completed with the modelled loop highlights the residues involved in the interaction interface; positively (blue) and negatively (red) charged residues are annotated and neutral residues are shown in purple. Inter-chain salt bridges that persist above the threshold are indicated by straight lines and those that do not persist by dotted lines. (*d*) The zebrafish PATZ1 dimerization interface is also shown as a split homodimer view for comparison. Numbering refers to the crystal structure files.

Sequence alignment shows that while the length of the A2/B3 loop is conserved in all PATZ1 orthologues, it is conspicuously absent in those from fish and amphibians (Fig. 1 ▸ *a*). The mammalian A2/B3 loop could be an evolutionarily acquired insertion sequence encoding an intrinsically disordered loop (IDL; Fukuchi *et al.*, 2006 ▸). To study the structure of this region in detail, we solved the structure of the zebrafish PATZ1 BTB domain to a resolution of 1.8 Å (Figs. 2 ▸ *c* and 2 ▸ *d*). The zebrafish PATZ1 BTB domain was expressed from a construct encoding amino acids 1–135 preceded at the N-terminus by 20 amino acids comprising a His tag and an HRV-3C protease digestion site. The 20 amino acids at the N-terminus and the first ten residues of the zebrafish PATZ1 BTB domain are not visible in the electron-density map. In addition, seven amino acids belonging to the A2/B3 loop are also missing from the electron density. When excluding the central loop, the murine and zebrafish sequences share 83.7% identity, whilst the structures can be superimposed with an r.m.s.d. of 0.62 Å (Fig. 2 ▸ *e*). The zebrafish BTB domain also has the same quaternary structure as the mouse BTB domain: a strand-exchange homodimer. Owing to the absence of the long disordered A2/B3 loop, the zebrafish PATZ1 BTB domain is structurally more similar to other ZBTB proteins than the mammalian PATZ1 BTB domain. The zebrafish PATZ1 BTB domain also has a seven-amino-acid sequence between A2 and B3 for which no discernible electron density could be found. This loop was modelled using *ModLoop* (Fiser *et al.*, 2000 ▸; Fiser & Sali, 2003 ▸; Fig. 3 ▸ *d*).

The human and mouse PATZ1 BTB domains have a single amino-acid difference (T91A). To detail the possible structure and dynamic behaviour of the A2/B3 loop in human PATZ1 BTB, we performed molecular modelling and molecular-dynamics simulations (MD). MD simulations lasting 200 ns indicated that while the overall dimer forms a stable structure, the A2/B3 loop region is uniquely flexible. The simulations also suggest that a new β-strand could form within this loop (Figs. 2 ▸ *f* and 3 ▸ *c*) and that at least ten amino acids within the modelled loop contribute to the homodimerization interface (representing 17.5% of the total interface of 57 amino acids). Interestingly, the β-strand that is generated in the simulations contains the threonine residue that is the only amino acid that differs between the human and mouse BTB domains. Other BTB domains such as LRF and MIZ1 also contain flexible loops in this region (Stogios *et al.*, 2007 ▸; Stead *et al.*, 2007 ▸). In the case of MIZ1, the A2/B3 region mediates tetramerization of its BTB domain, whilst using size-exclusion chromatography (Supplementary Fig. S1) we found no evidence for such oligomerization in mouse or zebrafish PATZ1.

### 3.2. A highly charged and dynamic surface contributes to the homodimerization interface of the PATZ1 BTB domain   

The dimerization interfaces of the mouse and zebrafish PATZ1 BTB domains are very similar. Using the *PIC* tool (Tina *et al.*, 2007 ▸), we determined that their crystal structures contain a single structurally corresponding salt bridge (Arg47–Glu75 in the mouse protein and Arg36–Glu64 in the zebrafish protein; the residue numbering refers to the crystal structures), in addition to the residues engaged in inter-chain hydrophobic interactions and hydrogen bonds (Figs. 3 ▸ *a* and 3 ▸ *b*). The interfaces retrieved from *PDBePISA* (Krissinel & Henrick, 2007 ▸) contain four basic and five acidic residues for the murine protein and four basic and three acidic residues for the zebrafish protein (Table 2 ▸).

In order to understand the dynamics of these interfaces, we assessed the number of contact-forming residues in the energy-minimized modelled BTB domains (Figs. 2 ▸ *f*, 3 ▸ *c* and 3 ▸ *d*). Using the *VMD* tools, we found a dramatic increase in the number of charged residues (mostly negative) that participate in interface contacts (marked with asterisks in Table 2 ▸). To understand the stability of these contacts, we assessed those that persist above a threshold value (15%) during the lifetime of the MD simulation (Figs. 3 ▸ *c* and 3 ▸ *d*). During the simulation, the A2/B3 loop region significantly contributes to the interface in both models, resulting in flexibility of the salt bridges that form between a single charged amino acid from one monomer and multiple opposite charged amino acids from the opposite monomer. While Arg47 of mouse PATZ1 BTB is only engaged with Glu75 in the crystal structure, MD show that it can contact a broader number of charged residues, including those from the flexible loop (Asp50, Asp84 and Asp89; Supplementary Fig. S6). We also find that two of the three degron residues (annotated in Figs. 1 ▸ *b* and 2 ▸ *f*) that are predicted to play a role in BTB heterodimer degradation participate in the interaction interface of both BTB homodimer structures.

### 3.3. Co-repressor binding modalities are not conserved in different BTB domains   

**Figure 4:**
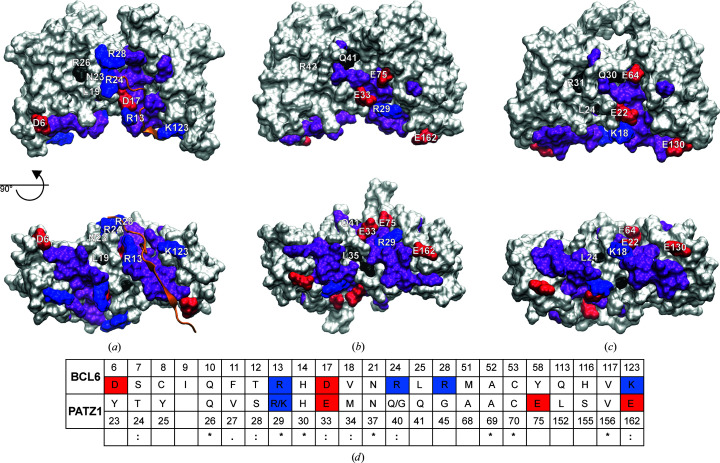
Comparison of the lateral grooves of PATZ1 and BCL6 BTB domains. The structures of the BCL6 (PDB entry 1r2b) (*a*) and the energy-minimized modelled mouse (*b*) and zebrafish (*c*) PATZ1 BTB domains are shown in surface representation viewed from the front and bottom. The surface area of the residues in the lateral groove of the BCL6 BTB domain is buried upon formation of the BCL6–SMRT complex (Ahmad *et al.*, 2003 ▸). The SMRT peptide binding to the BCL6 lateral groove is shown in cartoon representation in orange. All residues in the lateral groove are labelled for one monomer. Colours indicate residue type: positively charged, blue; negatively charged, red. All other residues in this region are coloured purple. The positions of the mutations that affect the binding to the co-repressor peptides in BCL6 and in PATZ1 are indicated in black (references are given in the text). A sequence alignment of BCL6 and PATZ1 BTB residues located in the lateral groove region is shown in (*d*). Residues are numbered according to the BCL6 and mouse PATZ1 structure files, with the charged residues coloured as in (*a*)–(*c*). Apart from positions 29 and 40, where the two alternatives are indicated, mouse and zebrafish PATZ1 contain the same residues in these structurally corresponding positions. Although the residue conservation for BCL6 and PATZ1 in this region is low, SMRT/NCOR peptides are predicted to bind the BTB domain of PATZ1 in the same region.

BTB domains have been shown to interact with the co-repressor proteins NCOR1, SMRT and BCOR (Huynh & Bardwell, 1998 ▸; Wong & Privalsky, 1998 ▸; Melnick *et al.*, 2002 ▸; Huynh *et al.*, 2000 ▸). Co-repressor binding to the BCL6 BTB domain requires dimerization because the interaction interface (lateral groove) is formed by residues in both monomers. 23 residues from each BCL6 monomer contribute to this interface (Ahmad *et al.*, 2003 ▸; Fig. 4 ▸). Additionally, four residues, when mutated (L19S, N23H and L25S/R26L), interfere with co-repressor binding by preventing homodimerization of the BTB domain (Huynh & Bardwell, 1998 ▸; Ghetu *et al.*, 2008 ▸; Granadino-Roldán *et al.*, 2014 ▸). The PATZ1 BTB domain has also been shown to bind to NCOR1, suggesting that a similar lateral groove may be mediating this interaction (Bilic *et al.*, 2006 ▸). In fact, when the residues corresponding to L19S, N23H and L25S/R26L in BCL6 were mutated in the PATZ1 BTB domain (L27S, Q33S and R34L), it also failed to bind NCOR1 (Bilic *et al.*, 2006 ▸).

Even though BCL6 and PATZ1 are structurally very similar, their corresponding co-repressor binding interface sequences are not conserved (Fig. 4 ▸ *d*). To examine the structural similarity between the PATZ1 and BCL6 BTB domains, we calculated the r.m.s.d. (1.56 Å) between individual monomers. The structural similarity between BCL6 and PATZ1 was more evident when the flexible PATZ1 loop was excluded (r.m.s.d. of 1.23 Å). Comparison of the surface charge distributions of the two proteins indicates major differences (Supplementary Fig. S5). Specifically, the BCL6 lateral groove contains a high density of positively charged amino acids that interact with the co-repressors (the interaction with SMRT is shown in Fig. 4 ▸ *a*). Surprisingly, the surface of PATZ1 corresponding to the BCL6 lateral groove did not contain as many basic residues (Fig. 4 ▸ *d* and Supplementary Fig. S5). In fact, this region of mouse and zebrafish PATZ1 is highly conserved (91% identical) and contains more acidic amino acids.

The presence of alternatively charged residues in the lateral groove of PATZ1 may indicate that its interaction with co-repressors may be through a different mode compared with BCL6. In this regard, the lateral groove of the PATZ1 BTB domain is more similar to that of LRF compared with BCL6 (Stogios *et al.*, 2007 ▸). We also find that mouse PATZ1 residue Asp50 (Supplementary Fig. S5) is part of the charged pocket that is conserved between BCL6, LRF and PLZF (Stogios *et al.*, 2007 ▸). Residue Asp50 in mouse PATZ1, corresponding to Asp39 in zebrafish and to Asp33 in BCL6, is absolutely conserved in all ZBTB proteins and happens to be the previously mentioned second degron residue (Fig. 1 ▸). The charged pocket that is formed by the participation of Asp50 residues from both monomers has been suggested as an alternative region for ligand binding (Supplementary Fig. S5; Melnick *et al.*, 2002 ▸).

## 4. Discussion   

A role for PATZ1 has been demonstrated in various malignancies such as thyroid and testicular cancer (Fedele *et al.*, 2008 ▸, 2017 ▸; Valentino, Palmieri, Vitiello, Pierantoni *et al.*, 2013 ▸; Chiappetta *et al.*, 2015 ▸; Vitiello *et al.*, 2016 ▸; Monaco *et al.*, 2018 ▸). The interaction between PATZ1 and the tumour suppressor p53 (Valentino, Palmieri, Vitiello, Pierantoni *et al.*, 2013 ▸; Valentino, Palmieri, Vitiello, Simeone *et al.*, 2013 ▸; Chiappetta *et al.*, 2015 ▸; Keskin *et al.*, 2015 ▸) mediated by a motif in the zinc-finger DNA-binding domain rather than the BTB domain also links this protein to cancer. Chromosome 22-specific inversions that translocate the transcription factor EWSR1 with PATZ1 have been observed in various sarcomas. While EWSR1 translocates with ETS family proteins in Ewing’s sarcoma, it potentially encodes two fusion proteins EWSR1-PATZ1 and PATZ1-EWSR1, the latter of which will contain the BTB domain (Siegfried *et al.*, 2019 ▸; Bridge *et al.*, 2019 ▸; Chougule *et al.*, 2019 ▸; Sankar & Lessnick, 2011 ▸; Mastrangelo *et al.*, 2000 ▸; Im *et al.*, 2000 ▸). The dimerization and co-repressor interaction properties of PATZ1 identified in this study may shed light on the mechanism of these sarcomas.

The gene targets of PATZ1 have not been extensively identified. ChIP-Seq and RNASeq experiments have identified 187 putative targets (Encode Project Consortium, 2012 ▸; Keskin *et al.*, 2015 ▸). Of these targets, roughly half were upregulated and half were downregulated in the absence of PATZ1 expression. How many of these genes are direct targets and how many require the BTB domain for regulation is not known. The current study only highlights structural motifs that are likely to play a role in the interaction of the PATZ1 BTB domain with co-repressor proteins. Yet other interactions may be involved in the potential role of PATZ1 in gene upregulation.

Our present study identifies important structural features of the PATZ1 BTB domain. One unique feature of BTB domains is their ability to form homodimers as well as heterodimers as a result of their close structural homology. Dimer formation is necessary for interaction with co-repressor proteins for both PATZ1 and BCL6 (Bilic *et al.*, 2006 ▸). A lateral groove that BCL6 uses to bind co-repressors is structurally conserved in PATZ1 (Fig. 4 ▸). However, the discrepancy in charged amino acids in this groove (Supplementary Fig. S5) may indicate altered binding modalities and/or affinities for co-repressors. In fact, a structurally conserved charged pocket has previously been hypothesized to be involved in ligand interaction (Stogios *et al.*, 2007 ▸). While this charged pocket is surface-exposed in BCL6, LRF and PLZF structures, the PATZ1 A2/B3 loop could dynamically gate this site, potentially regulating ligand interaction.

Another feature that is common to PATZ1 and BCL6 is their BTB domain-mediated localization to nuclear speckles (Huynh *et al.*, 2000 ▸; Fedele *et al.*, 2000 ▸; Franco *et al.*, 2016 ▸). While PATZ1 interacts with the nuclear speckle-resident ubiquitin ligase RNF4, whether potential post-translational modifications owing to this interaction affect its stability is not known (Pero *et al.*, 2002 ▸). A recent study identified three BTB domain degron residues that are surface-exposed preferentially in heterodimers (Mena *et al.*, 2018 ▸). The targeting of these degrons by ubiquitin ligases mediates the proteasome-dependent degradation of heterodimers over homodimers. It is not known whether this is a generalizable feature of BTB domains. Consistent with the stability of homodimers, in the current study we find that PATZ1 buries two of these three degron residues in the protein globular structure (Fig. 2 ▸ *f*).

The MD simulation shows that alternative contacts are possible for several charged residues at the homodimer interface (Table 2 ▸). Because a single charged residue can contact more than one oppositely charged residue (Fig. 3 ▸), the dimer interface may be more resilient to the disruption of single contacts. Energetically, the presence of alternative salt bridges may be necessary to accommodate the flexibility of the central loop whilst retaining the stability of the dimerization interface. Nevertheless, there are an exceptional number of unpaired charged surface residues in the murine PATZ1 BTB domain. Some proteins, for example calmodulin, with large net surface charges are known to modulate their environment by redistributing nonspecific ions in the surrounding medium (Aykut *et al.*, 2013 ▸). Such modes of action are utilized to shift the population of the available conformational states, leading to fine-tuned functions.

BTB domains are attractive targets for anticancer compounds. Compounds that prevent homodimerization or result in the degradation of BCL6 (Kerres *et al.*, 2017 ▸) have been shown to have highly effective cytotoxic activity in B-cell lymphomas. Because the BTB domains of ZBTB family proteins all share the same fold, compound specificity requires the targeting of unique features. The residues in the A2/B3 loop of PATZ1, which are unique among the ZBTB proteins, are potentially a specific target for this protein (Fig. 1 ▸). The structure of the PATZ1 BTB domain reported in this study will aid in the development of therapeutics for those human malignancies that involve PATZ1 and the testing of the specificity of compounds targeting other BTB domains.

## Supplementary Material

PDB reference: mouse PATZ1 BTB domain, 6guv

PDB reference: zebrafish PATZ1 BTB domain, 6guw

Supplementary Figures. DOI: 10.1107/S2059798320005355/ni5006sup1.pdf

## Figures and Tables

**Table 1:** Data-collection and refinement statistics Values in parentheses are for the highest resolution shell.

	Mouse PATZ1 BTB	Zebrafish PATZ1 BTB
Data-collection statistics		
Wavelength (Å)	0.97625	0.9795
Space group	*P*4_1_2_1_2	*P*3_1_21
*a*, *b*, *c* (Å)	43.23, 43.23, 162.95	43.08, 43.08, 123.64
α, β, γ (°)	90, 90, 90	90, 90, 120
Resolution (Å)	43.23–2.29 (2.37–2.29)	41.8–1.8 (1.9–1.8)
No. of unique reflections	7573 (724)	12786 (1120)
*R*_merge_	0.05 (0.67)	0.01 (0.49)
〈*I*/σ(*I*)〉	9.91 (1.39)	19.96 (1.27)
Completeness (%)	99.58 (99.18)	98.41 (87.99)
Multiplicity	2.0 (2.0)	2.0 (1.8)
Refinement statistics		
Resolution (Å)	41.79–2.29	41.2–1.8
No. of reflections (observed/*R*_free_)	7549/417	12785/571
*R*_work_/*R*_free_	0.209/0.245	0.215/0.224
No. of atoms		
Total	1123	993
Protein	1057	940
Water	66	53
Average *B*, all atoms (Å^2^)	54.3	50
R.m.s. deviations		
Bond lengths (Å)	0.002	0.007
Bond angles (°)	0.433	0.965
PDB code	6guv	6guw

**Table 2:** Inter-chain salt bridges in the dimerization interface of the BTB domain of PATZ1 Residue numbers refer to the crystal structures.

BTB protein	PDB code	Inter-chain salt bridges	Frequency in MD (%)	Intra-monomer salt bridges	
				Intra-interface	All protein
Mouse PATZ1	6guv	Glu75–Arg47 (crystal only)	0	Asp50–Arg64	Asp127–Arg42
		Asp50–Arg47 (*B*–*A*)†	64.84	Asp124–Arg137	Glu111–Arg54†
		Asp84–Arg47†	7.54 (*BA*)–32.77 (*AB*)	Asp84–Arg64†	Glu111–Arg110 (*B*–*B*)†
		Asp89–Arg47 (*A*–*B*)†	11.14	Asp94–Arg110†	Asp127–Lys43†
		Glu158–Arg29†	32.32 (*AB*)–76.82 (*BA*)	Glu72–Arg154†	Glu143–Lys120†
				Asp50–Arg110 (*A*–*A*)†	Asp57–Arg54†
				Asp89–Arg64 (*A*–*A*)†	Asp57–Lys120†
				Asp89–Arg110†	Asp124–Lys120†
				Asp89–Arg47 (*B*–*B*)†	Glu58–Arg110 (*A*–*A*)†
				Asp127–Arg133†	Glu58–Lys120†
				Glu33–Arg29†	Glu58–Lys43 (*B*–*B*)†
				Glu75–Arg64 (*A*–*A*)†	Glu139–Lys120†
				Glu139–Arg137†	Asp94–Arg64 (*A*–*A*)†
				Glu143–Arg137†	
				Glu158–Arg154†	
				Glu162–Lys165†	
Zebrafish PATZ1	6guw	Glu64–Arg36	77.42 (*BA*)–85.7 (*AB*)	Asp92–Arg105	Asp95–Arg31
		Asp39–Lys53 (*A*–*B*)†	5.44	Asp33–Arg36†	Glu61–Arg122
		Asp74–Lys78†	5.89 (*AB*)	Asp39–Lys53†	Glu107–Lys88
		Glu22–Arg122 (*A*–*B*)†	12.84	Asp39–Lys78 (*A*–*A*)†	Asp33–Lys32†
		Glu72–Arg36 (*A*–*B*)†	93.06	Asp73–Lys53 (*A*–*A*)†	Asp92–Arg91†
		Glu126–Lys18†	19.08 (*AB*)–28.42 (*BA*)	Asp92–Lys101 (*A*–*A*)†	Asp92–Lys88†
				Asp95–Lys101†	Asp95–Arg91†
				Glu22–Lys18†	Asp95–Lys32†
				Glu22–Arg122 (*A*–*A*)†	Glu46–Arg43†
				Glu72–Lys53†	Glu46–Arg91 (*A*–*A*)†
				Glu107–Arg105†	Glu46–Lys88†
					Glu47–Arg91†
					Glu47–Lys88†
					Glu79–Arg43†
					Glu79–Lys78†
					Glu81–Arg43†
					Glu111–Lys88†
					Glu126–Arg122 (*A*–*A*)†

† Added with MD simulation of the modelled structures.
