# Supplementary material for: Structural analysis of the PATZ1 BTB domain homodimer
Source: Acta Crystallogr D Struct Biol. 2020 May 29;76(Pt 6):581–93. doi: 10.1107/S2059798320005355 (PMC7271949; doi:10.1107/S2059798320005355)
Supplement: Supplementary file 1 [file d-76-00581-sup1.pdf]

STRUCTURAL
BIOLOGY

Volume 76 (2020)

Supporting information for article:

Structural analysis of the PATZ1 BTB domain homodimer

Sofia Piepoli, Aaron Oliver Alt, Canan Atilgan, Erika Jazmin Mancini and Batu Erman

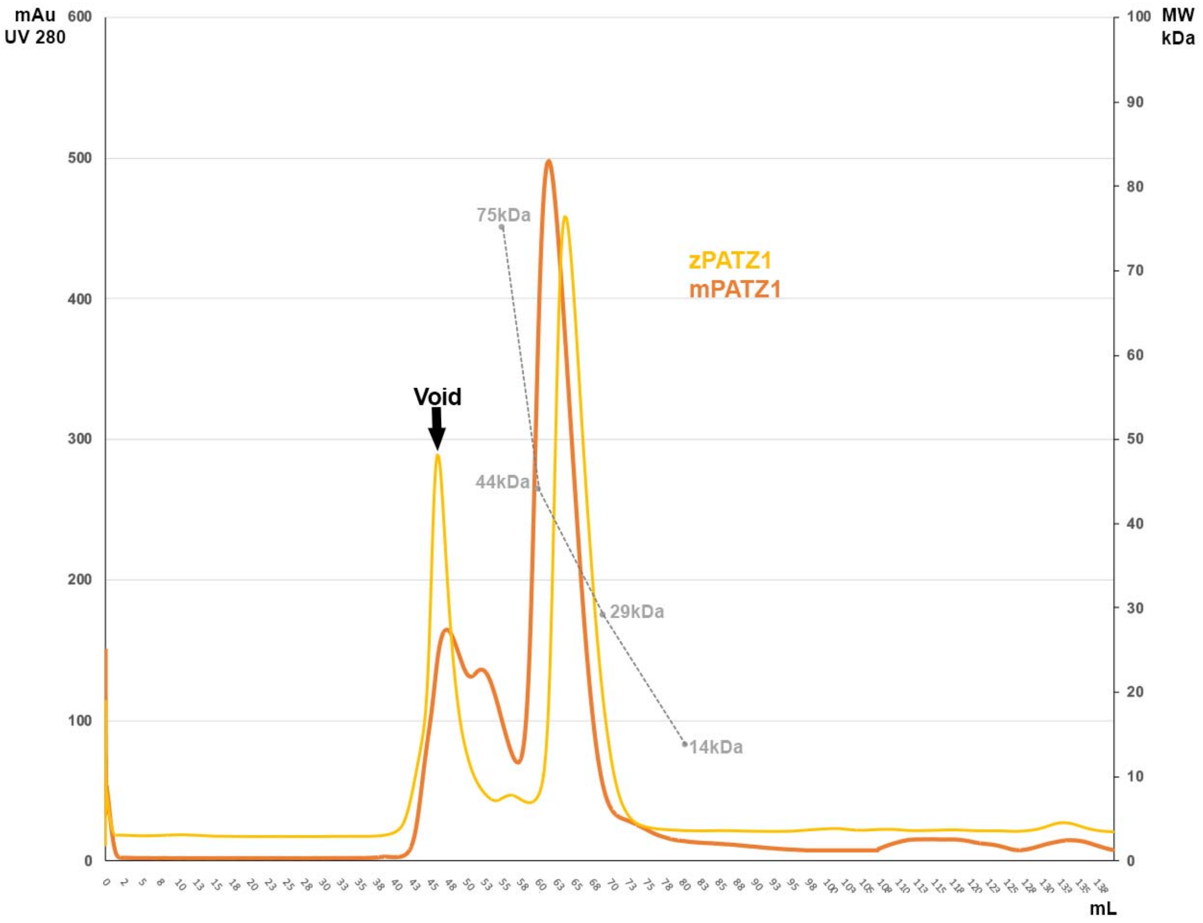

Figure S1 Size exclusion chromatography (SEC) indicates that both murine and zebrafish PATZ1 BTB domains form mainly homodimers in solution. The chromatographic profiles and elution volumes of mouse (m) and zebrafish (z) PATZ1 BTB domains are plotted as a function of absorbance units at UV 280nm (left axis) in orange and yellow, respectively. A standard curve is plotted as a grey dotted line connecting the elution points of calibration markers run on the same column (right axis). For the constructs of the BTB domain of mPATZ1 (6GUV) and zPATZ1 (6GUW), the calculated molecular weight (MW) for the monomer is 18.7 kDa and 17.7 kDa, respectively. Thus, the elution points for both constructs peaking between the 29 and 44 kDa markers indicate the prevalence of dimeric conformations in solution for both proteins. Details for both purified protein constructs are elucidated in the Methods Section.

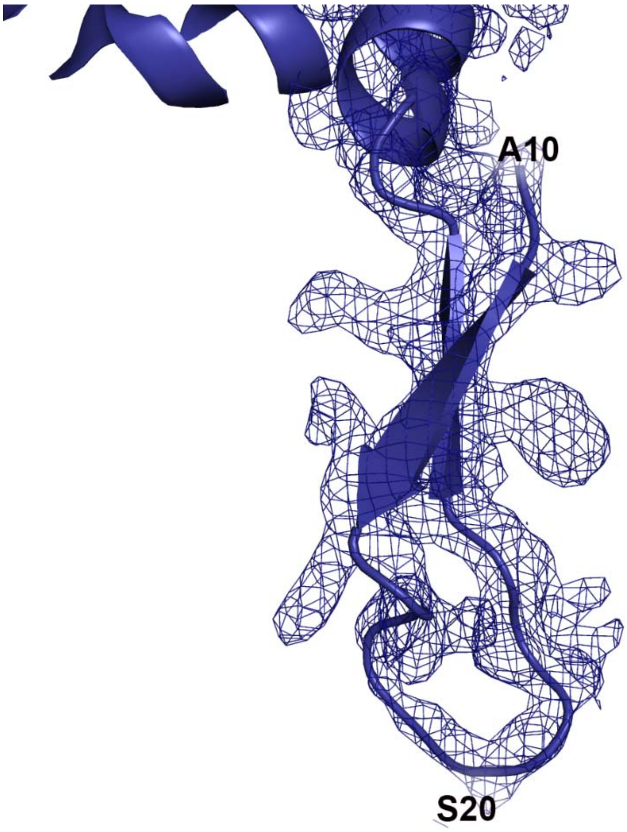

Figure S2 Enlargement of the N-terminal region of one monomer in the mouse PATZ1 BTB domain (6GUV). The electron density map is shown for the secondary structure elements β0 and β1.

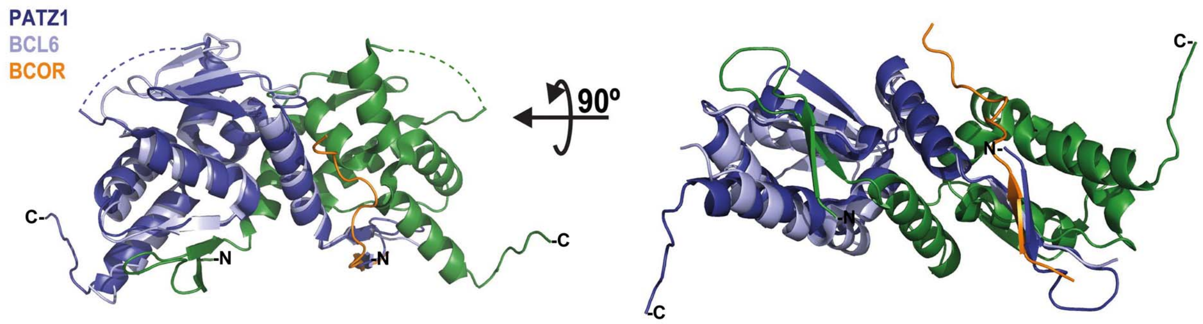

Figure S3 Superposition of the BCOR bound BCL6 BTB domain monomer structure with that of the mouse PATZ1 BTB domain dimer in front and side view. Two PATZ1 monomers comprising a homodimer are shown in green and blue, BCL6 monomer is shown in violet and BCOR co-repressor peptide is shown in orange. His-tag residues form an N-terminal β-strand (β0) of the PATZ1 BTB domain construct to mimic the β-sheet interaction of co-repressors with BCL6. The missing residues of murine PATZ1 A2/B3 loop are indicated by dotted lines.

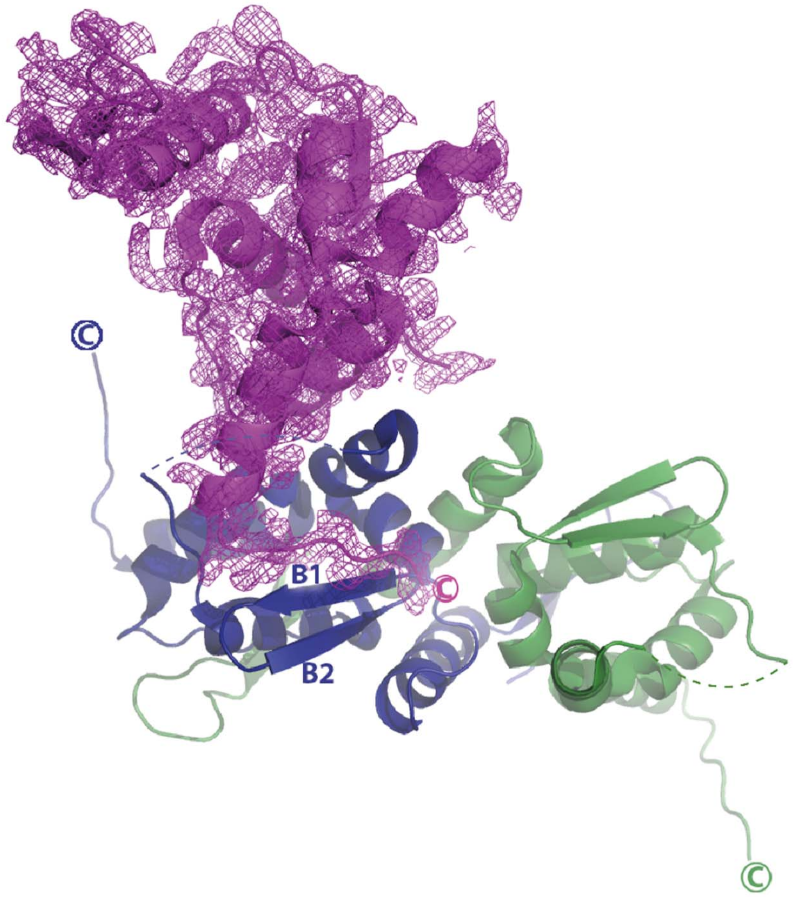

Figure S4 A C-terminal sequence from an adjacent molecule mimics the B3 β-strand to stabilize the β-sheet formed by B1 and B2 strands in the mouse PATZ1 BTB domain. Two PATZ1 BTB monomers forming a homodimer in the asymmetric unit are shown in green and blue respectively (top view). A third monomer from an adjacent molecule (shown in magenta with additional electron density) contributes to the β-sheet formed by B1 and B2. The missing residues of murine PATZ1 A2/B3 loop are indicated by dotted lines.

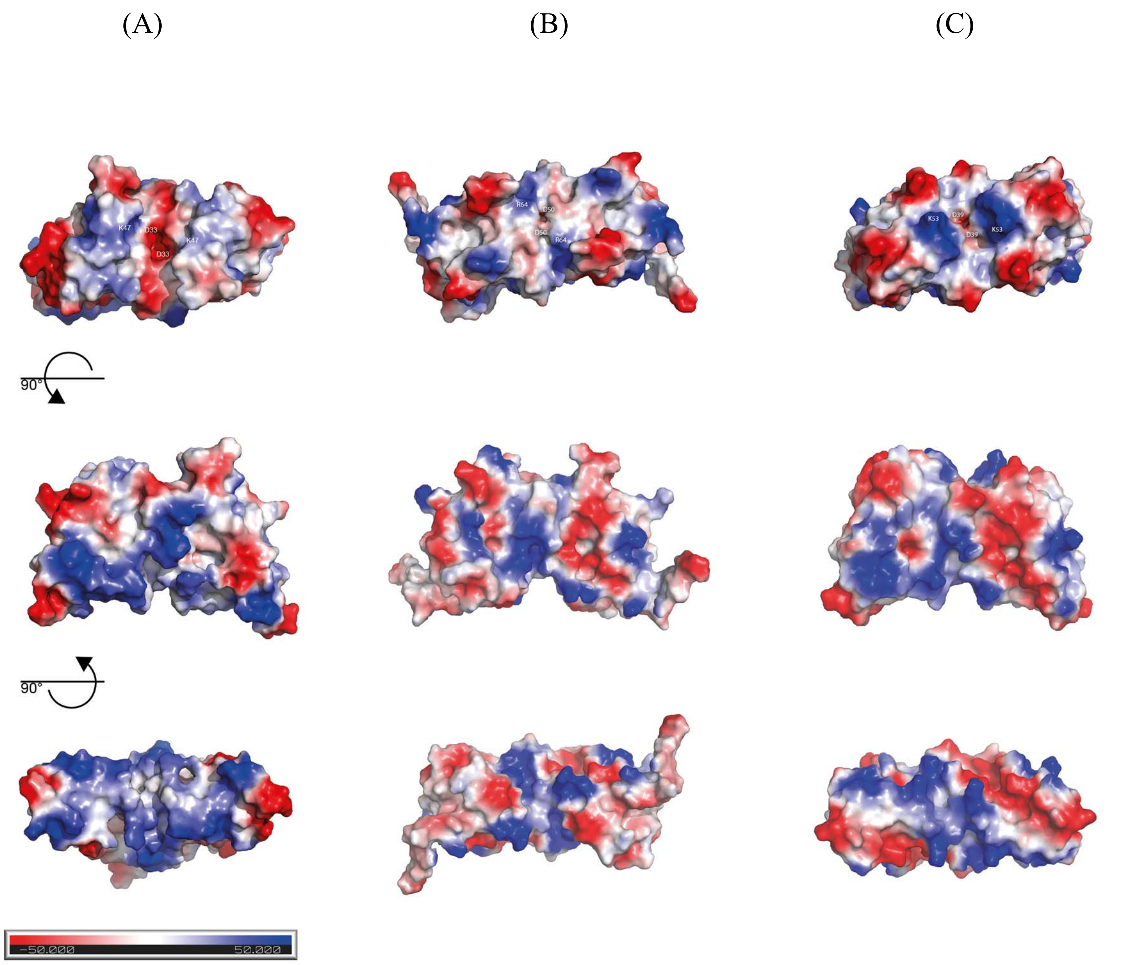

Figure S5 Comparison of the electrostatic surface potentials of PATZ1 and BCL6 BTB domains. From the central front view, two 90º rotations show top and bottom views of the electrostatic surface potential of the BTB domain of: **(A)** BCL6 (PDB entry 3E4U); **(B)** murine PATZ1 (6GUV); **(C)** zebrafish PATZ1 (6GUW). In the top view two conserved charged residues (negative and positive) that characterize the BTB domain charged pocket are indicated. Residues are numbered according to the BCL6 and PATZ1 structure files. The colors range from red (negative potential) to blue (positive potential) with white near neutral.

(A)

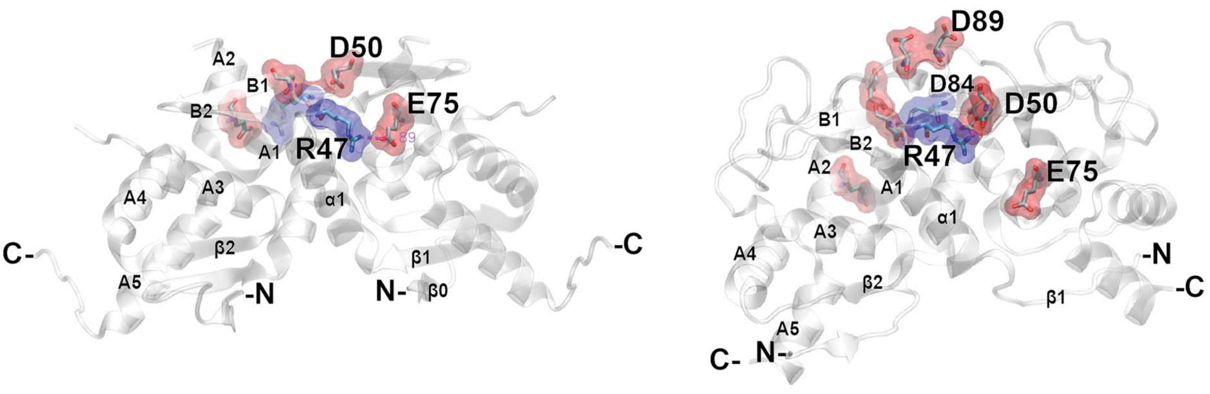

(B) (C)

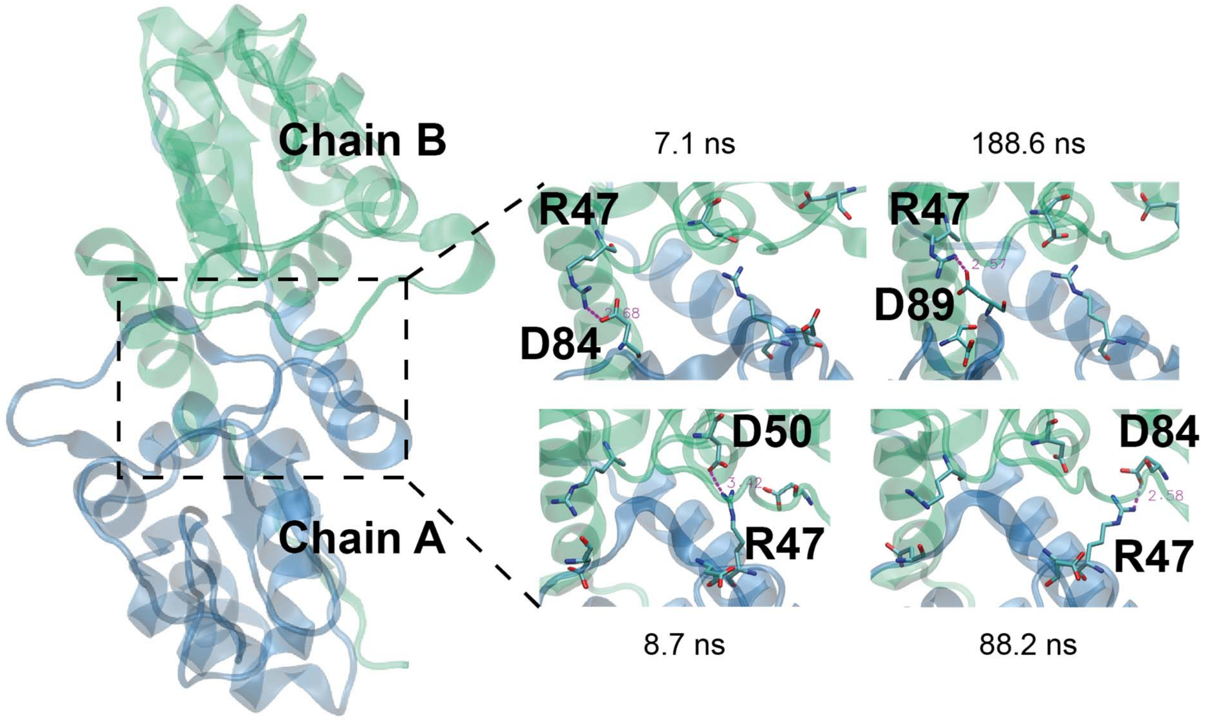

Figure S6 Molecular Dynamics (MD) Simulation shows that interface residues rearrange through alternative contacts. (A) The salt bridge between R47 and E75 in the crystal structure (6GUV) on the left is not retained in the energy minimized structure on the right (front view). (B) A partial dimer interface between the two monomers (Chain A and B) of the mouse PATZ1 BTB domain including the energy minimized modelled central loop is shown in the dashed box (top view). (C) Alternative salt bridges (purple dotted lines, distance in Å) are shown at different time frames of the MD simulation.
